# The *PIK3CA* E542K and E545K mutations promote glycolysis and proliferation via induction of the β-catenin/SIRT3 signaling pathway in cervical cancer

**DOI:** 10.1186/s13045-018-0674-5

**Published:** 2018-12-14

**Authors:** Wei Jiang, Tiancong He, Shuai Liu, Yingying Zheng, Libing Xiang, Xuan Pei, Ziliang Wang, Huijuan Yang

**Affiliations:** 1 Department of Gynecological Oncology, Fudan University Shanghai Cancer Center, Fudan University, Shanghai, 200032 China; 2 Department of Cancer Institute, Fudan University Shanghai Cancer Center, Fudan University, Shanghai, 200032 China; 3 Department of Nuclear Medicine, Fudan University Shanghai Cancer Center, Fudan University, Shanghai, 200032 China; 4 Department of Oncology, Shanghai Medical College, Fudan University, 270 Dong’an Road, Shanghai, 200032 China

**Keywords:** *PIK3CA* E542K and E545K mutations, β-Catenin, SIRT3, Glycolysis, Cervical cancer

## Abstract

**Background:**

The study aims to present the effect of *PIK3CA* E542K and E545K mutations on glucose metabolism and proliferation and identify their underlying mechanisms in cervical cancer.

**Methods:**

The maximum standard uptake value (SUV_max_) of tumors was detected by^18^F-FDG PET/CT scan. In vitro, glycolysis analysis, extracellular acidification rate analysis, and ATP production were used to evaluate the impact of *PIK3CA* E542K and E545K mutations on glucose metabolism. The expression level of key glycolytic enzymes was evaluated by western blotting and immunohistochemical staining in cervical cancer cells and tumor tissues, respectively. Immunofluorescence analysis was used to observe the nuclear translocation of β-catenin. The target gene of β-catenin was analyzed by using luciferase reporter system. The glucose metabolic ability of the xenograft models was assessed by SUV_max_ from microPET/CT scanning.

**Results:**

Cervical cancer patients with mutant *PIK3CA* (E542K and E545K) exhibited a higher SUV_max_ value than those with wild-type *PIK3CA* (*P* = 0.037), which was confirmed in xenograft models. In vitro, enhanced glucose metabolism and proliferation was observed in SiHa and MS751 cells with mutant *PIK3CA*. The mRNA and protein expression of key glycolytic enzymes was increased. AKT/GSK3β/β-catenin signaling was highly activated in SiHa and MS751 cells with mutant *PIK3CA*. Knocking down β-catenin expression decreased glucose uptake and lactate production. In addition, the nuclear accumulation of β-catenin was found in SiHa cells and tumors with mutant *PIK3CA*. Furthermore, β-catenin downregulated the expression of SIRT3 via suppressing the activity of the SIRT3 promotor, and the reduced glucose uptake and lactate production due to the downregulation of β-catenin can be reversed by the transfection of SIRT3 siRNA in SiHa cells with mutant *PIK3CA*. The negative correlation between β-catenin and SIRT3 was further confirmed in cervical cancer tissues.

**Conclusions:**

These findings provide evidence that the PI3K E542K and E545K/β-catenin/SIRT3 signaling axis regulates glucose metabolism and proliferation in cervical cancers with *PIK3CA* mutations, suggesting therapeutic targets in the treatment of cervical cancers.

**Trial registration:**

FUSCC 050432–4-1212B. Registered 24 December 2012 (retrospectively registered).

**Electronic supplementary material:**

The online version of this article (10.1186/s13045-018-0674-5) contains supplementary material, which is available to authorized users.

## Background

Cervical cancer remains one of the most common gynecological malignancies, accounting for more than half a million new cases and leading to approximately 266,000 deaths annually worldwide [1]. The clinical outcome of early-stage cervical cancer has been improved due to progress in diagnosis and treatment, whereas the prognosis of advanced and metastatic cervical cancer is still unsatisfactory. Consequently, increasing studies have focused on exploring the molecular characteristics of cervical cancer to identify new therapeutic targets to improve the prognosis of this disease.

The phosphatidylinositol 3-kinase (PI3K) pathway is recognized as one of the most activated signaling pathways in human cancers. Molecular aberrations of the PI3K pathway drive tumorigenesis and promote various biological processes, including cell proliferation, invasion and migration, differentiation, apoptosis, and glucose metabolism [2–4]. These alterations are mainly caused by *PIK3CA* amplification or mutation [5, 6] and *PTEN* loss [7]. *PIK3CA* mutation has been observed in various solid tumors and plays a crucial and intricate role in carcinogenesis and the development of malignant tumors [8, 9]. In cervical cancer, *PIK3CA* has been identified as one of the most commonly mutated genes, and the mutation rate ranges from 10 to 30% [10–12]. In a previous study, we demonstrated that E545K, E542K (helical domain), and H1047R (kinase domain) were the hotspots of *PIK3CA* mutation in cervical cancer. Different from breast cancer, the occurrence of E545K and E542K mutations is dramatically higher than that of H1047R in cervical cancer [13]. *PIK3CA* mutations in helical and kinase domains exhibit distinct biological and clinical characteristics due to the activation of different signaling pathways. The P110a E545K mutation activates the AKT pathway via interacting with IRS1 instead of the regulatory subunit p85 compared with H1047R mutation [14]. As a result, it is necessary to elucidate the regulatory function of the mutated hotspots E542K and E545K in cervical cancer.

Cancer cells have to readjust their energy metabolism to sustain the uncontrolled proliferation under conditions of complete or deficient nutrients, namely, the Warburg effect, suggesting that cancer cells preferentially utilize aerobic glycolysis to support energetic demands for rapid growth, even in the presence of oxygen [15]. Consequently, some aberrant activation of oncogenic pathways regulates glucose uptake and catabolism. Recent studies suggest that glucose metabolism is mainly reprogrammed by mutations in TP53, MYC, Ras-related oncogenes, and the LKB1-AMP kinase (AMPK) and PI3 kinase (PI3K) signaling pathways [16]. Oncogenic PI3K activation regulates glucose metabolism by regulating a series of downstream signaling effectors. The activation of AKT is essential to stimulate aerobic glycolysis to provide energy for cancer cells [17–19]. The activation of the PI3K/AKT pathway promotes the translocation of glucose transporter 4 (GLUT4) to the plasma membrane in response to insulin stimulation [18]. Furthermore, colorectal cancer cells with *PIK3CA* mutations exhibit increased proliferation by glutamine dependence [20]. However, the role of mutant *PIK3CA* on metabolism in cervical cancer is less researched.

In the present study, we demonstrate that *PIK3CA* mutations promoted glucose metabolism and cervical cancer cell proliferation in vivo and in vitro. Mechanistically mutant PI3K enhanced the expression of β-catenin by activating AKT/GSK3β signaling and promoted the nuclear translocation of β-catenin to transcriptionally decrease the expression of SIRT3, which is a negative regulator of glucose metabolism. These results indicate that *PIK3CA* E542K and E545K mutations play a positive role in regulating glucose metabolism by activating β-catenin/SIRT3 signaling pathways in cervical cancer.

## Methods

### Patients and specimens

The present study was approved by the Ethics Committee at Fudan University Shanghai Cancer Center (FUSCC 050432–4-1212B) and conducted in accordance with the approved guidelines. A total of 1015 cervical cancer patients were recruited from January 2010 to December 2014. Altogether, 990 cervical cancer patients were included in the present study, since the cDNA of 25 patients was exhausted. The following inclusion criteria were considered according to a previous publication [13]: pathologically confirmed primary cervical cancer, stage IB1-IIA2 according to the International Federation of Gynecology and Obstetrics (FIGO) staging system, and no neoadjuvant chemotherapy or radiation. Tumor tissues were collected during radical hysterectomy procedures. Informed consent was obtained from each patient prior to treatment.

### Determination of *PIK3CA* mutation status

The *PIK3CA* mutation status of 990 patients was detected by Sanger sequencing. A total of 771 patients were recruited from January 2010 to December 2012, and their mutation statuses were reported in a previous study [13]. An additional 219 patients were recruited from January 2013 to December 2014, and the mutation status of these patients was determined as previously described [13]. There was no difference of the mutation status between the 771 patients and the 219 patients.

### Whole-body ^18^F-FDG PET/CT scan

Among the 990 patients, PET/CT scan was performed on 52 patients through clinical data retrieval, including 46 patients with wild-type *PIK3CA* and 6 patients with *PIK3CA* E542K and E545K mutations. The SUV_max_ value of the cervical tumors was detected by PET/CT scan and used to compare the level of glucose metabolism in cervical tumors between patients with wild-type and mutant *PIK3CA*.

### Cell lines and cell culture

A previous study demonstrated that *PIK3CA* mutations more commonly occurred in squamous carcinoma of the cervix (SCC) [13]. Human SCC cell lines SiHa and MS751 with wild-type *PIK3CA* were obtained from American Type Culture Collection (ATCC). Both cell lines were cultured in DMEM supplemented with 10% FBS, 100 U/ml penicillin, and 100 mg/ml streptomycin and incubated under 37 °C with 5% CO_2_.

### Plasmid and viral transfection

The plasmid pENTER-*PIK3CA* E542K and E545K containing the Flag tag and the plasmid with short hairpin RNA (shRNA) against the open reading frame of β-catenin mRNA (positions 37–65,5-GCCATGGAACCAGACAGAAA-3) were purchased from Hanyin Biotechnology Limited Company (Shanghai). Then, the plasmid was subcloned into lentivirus vector pCDH-CMV-MCS-EF1-PURO to construct a recombinant plasmid. Similarly, the control vector was constructed by inserting oligonucleotides encoding short hairpin RNA against green fluorescence protein mRNA (shGFP) into the pLKO.1 vector.

Approximately 30 × 10^4^ SiHa and MS751 cells were seeded onto 6-well plates. The MOI value was set as 10 according to a preliminary experiment. The medium was replaced with 2 ml of virus solution, prepared in accordance with the best MOI value, 30 μl of virus (1 × 10^8^ TU/ml) mixed with 10 μl of polybrene (10 μg/ml), and the rest of the volume was supplemented with culture medium without serum. After 8 h, the medium in the plate was changed to medium with serum. The cells were transferred to 60-mm cell dishes after 48 h and prepared for PURO selection.

The concentration of PURO was determined according to the standard that all cells without virus infection were dead after exposure to a certain concentration of PURO, which was set as 2.5 mg/ml for SiHa and MS751 cells. The cells were treated with PURO for 48 h, and then, the cell medium with PURO was changed to normal medium or medium with PURO for a second round of selection according to the state of the cells. Generally, PURO selection was performed at least twice, and the surviving cells were prepared for identification by Sanger sequencing or western blotting to determine protein expression.

### Proliferation assay

The cells were seeded onto 96-well plates with 1 × 10^3^cells/well. Then, 10 μl CCK-8 solution was dissolved in 90 μl of DMEM medium and subsequently added to each well every day for a total of 7 days. The plates were incubated for 2 h, and the absorbance value was measured at a wavelength of 450 nm.

### Glycolysis analysis

Glucose Uptake Colorimetric Assay Kits (BioVision) were used to detect the glucose uptake in SiHa and MS751 cells with wild-type and mutant *PIK3CA*, according to the manufacturer’s protocols. Approximately 3 × 10^4^cells/well were seeded onto 6 wells of a 96-well plate. The medium was replaced with 100 μl of KRPH buffer the next day. Then, 10 μl of 2-DG was added to 3 wells and incubated for 20 min under 37 °C. Subsequently, 80 μl of Extraction Buffer was added and incubated for 40 min under 85 °C, followed by incubation for 5 min on ice, and OD detection was performed after adding 10 μl of neutralization solution. Lactate Colorimetric Assay Kits (Biovision) were used to evaluate the level of lactate production, according to the manufacturer’s protocols. A total of 50 × 10^4^ cells was collected in 1.5-ml EP tubes, and 100 μl of pre-cooling lactate assay buffer was added. After 15 min, the supernatant was collected after centrifugation at 12,000r for 5 min and prepared for OD450 nm.

### Extracellular acidification rate analysis

The Seahorse XF Cell Mito stress test kit was used to determine the extracellular acidification rate (ECAR) according to the manufacturer’s instructions. Subsequently, 4 × 10^4^ cells were seeded onto 96-well plates. The final ECAR values were obtained after normalization to the cell number.

### ATP (adenosine triphosphate) production analysis

The ENLITEN ATP Assay System (Promega; FF2000) was used to examine ATP production according to the manufacturer’s instructions. A diluted ATP standard was used to build a regression curve to calculate the ATP concentration of the samples. The relative ATP concentration was obtained after normalization to that of control cells.

### Western blot assay

Western blot was used to evaluate the protein expression level in the cells as previously described [21]. Antibodies against AKT, pAKT (Ser473 and Ser308), GSK3β, pGSK3β (Ser9), β-catenin, pβ-catenin (Thr41/Ser45), and SIRT3 were purchased from Cell Signaling Technology (CST), and antibodies against GLUT4 and LDHB (Lactate dehydrogenase B) were purchased from Proteintech. All primary antibodies were diluted 1:1000.

### Quantitative real-time PCR

TRIzol reagent (Invitrogen) was used for total RNA extraction. The cDNA was prepared by using the PrimeScript RT reagent kit (TAKARA) and subsequently used for real-time PCR analysis by using an ABI 7900HT Real-Time PCR system (Applied Biosystems). The primer sequences are listed in Additional file 1: Table S1. The following PCR program was used: 95 °C for 10 s, one cycle; 95 °C for 5 s, 62 °C for 31 s, 40 cycles; 2^−∆∆CT^ was used for the relative statistical analysis.

### Nuclear and cytoplasmic protein extraction

SiHa cells with PIK3CA WT E542K and E545K mutation were seeded onto 100-mm dishes at a density of 80–90%. The cells were scraped into 1.5-ml EP tubes after PBS washing. A Nuclear and Cytoplasmic Protein Extraction Kit (Beyotime Biotechnology, Shanghai, China) was used for nuclear and cytoplasmic protein extraction according to the manufacturer’s instructions (Beyotime Biotechnology, Shanghai, China). The obtained protein was prepared for protein quantification and western blot analysis.

### Immunohistochemical staining (IHC)

The protein expression in tumors from xenograft models and 60 patients (20 patients with wild-type *PIK3CA*, 20 patients with mutant *PIK3CA* E542K, and 20 patients with mutant *PIK3CA* E545K) was determined by IHC according to a previous publication [21]. Primary antibodies against the following proteins were used: P110a (dilution 1:50, Proteintech), AKT (dilution 1:200, CST), β-catenin (dilution 1:200, Proteintech), SIRT3 (dilution 1:100, CST), GLUT4 (dilution 1:50, Proteintech), and LDHB (dilution 1:1000, Proteintech). The secondary antibody was obtained from an IHC kit from Beijing CoWin Bioscience Co. Ltd. (Beijing, China). The staining was repeated by at least three performers who did not know others’ results and assessed by two pathologists blinded to the patients’ information. The final score was calculated as the staining intensity multiplied by staining area. The staining intensity and area of every slide were evaluated after observing five different visual fields by each pathologist. The staining intensity of tissues from patients was evaluated according to the standards shown in Fig. 6 (0 = no staining, 1 = weak staining, 2 = intermediate staining, and 3 = strong staining). The location of β-catenin was evaluated as follows: (1) membrane, if staining was mainly present at membrane but not at nucleus; (2) cytoplasm, if staining was predominant located at cytoplasm but not at nucleus; and (3) nucleus, if staining was present at the nucleus.

### Immunofluorescence assay

A total of 2 × 10^4^cells were seeded onto 24-well plates containing chamber slides. After attaching to the plate, the cells were fixed with 4% formaldehyde for 30 min. Then, 0.1% Triton 100 was used to permeabilize the plasma membrane for 30 min. The cells were subsequently washed three times with PBS for 5 min each, blocked with 5% BSA, and incubated in a primary antibody against β-catenin (dilution 1:50, Proteintech) under 4 °C overnight. After washing three times with PBS for 10 min each, the cells were incubated in secondary antibody (1:2000) for 1 h. The slides were counterstained with DAPI and stored under 4 °C in darkness. Images were captured by a Leica SP5 confocal fluorescence microscope.

### SiRNA interference

SiRNA against SIRT3, which was obtained from RuiBo Biotechnology Limited Company (Guangzhou), was transfected into SiHa cells by using FuGENE HD (Promega). The sequence was GTCCATATCTTTTTCTGTG.

### Luciferase assay

The SIRT3 promotor sequence was cloned into the pGL3-basic reporter gene vector. A plasmid with SIRT3 promotor was designed and constructed by Hanyin Biotechnology Limited Company (Shanghai). SiHa and MS751 cells with wild-type and mutant *PIK3CA* (8 × 10^3^ per well) were seeded onto 96-well plates, and then, the vector was transfected into cells with FuGENE HD (Promega). The luciferase reporter gene assays were performed by a luciferase assay system (Promega).

### Animal model

Altogether 1 × 10^7^cells (SiHa and MS751) mixed with 100 μl of PBS were injected into BALB/c-nu mice (female, 4 weeks of age; Shanghai SLAC Laboratory Animal Co., Ltd.). Each cell line was implanted into three mice. MicroPET/CT scanning was performed after 18 days. Then, the mice were raised until the 21st day. The tumors were surgically removed, fixed in 10% formalin, and subjected to routine histological examination. The tumor volume was calculated as the longest diameter × the shortest diameter^2^ × 0.5. All animal experiments were approved by the Institutional Animal Care and Use Committee of Fudan University and performed according to institutional guidelines.

### Statistical analysis

All experiments, except the animal experiments, were repeated three times. Two-tailed unpaired Student’s *t* tests and one-way ANOVA (analysis of variance) were used to evaluate the differences between two groups. GraphPad Prism 6 software (San Diego, CA) was used for the statistical analysis. The statistical significance is defined as *P* < 0.05.

## Results

### *PIK3CA* E542K and E545K mutations were prominent in cervical cancer

**Fig. 1:**
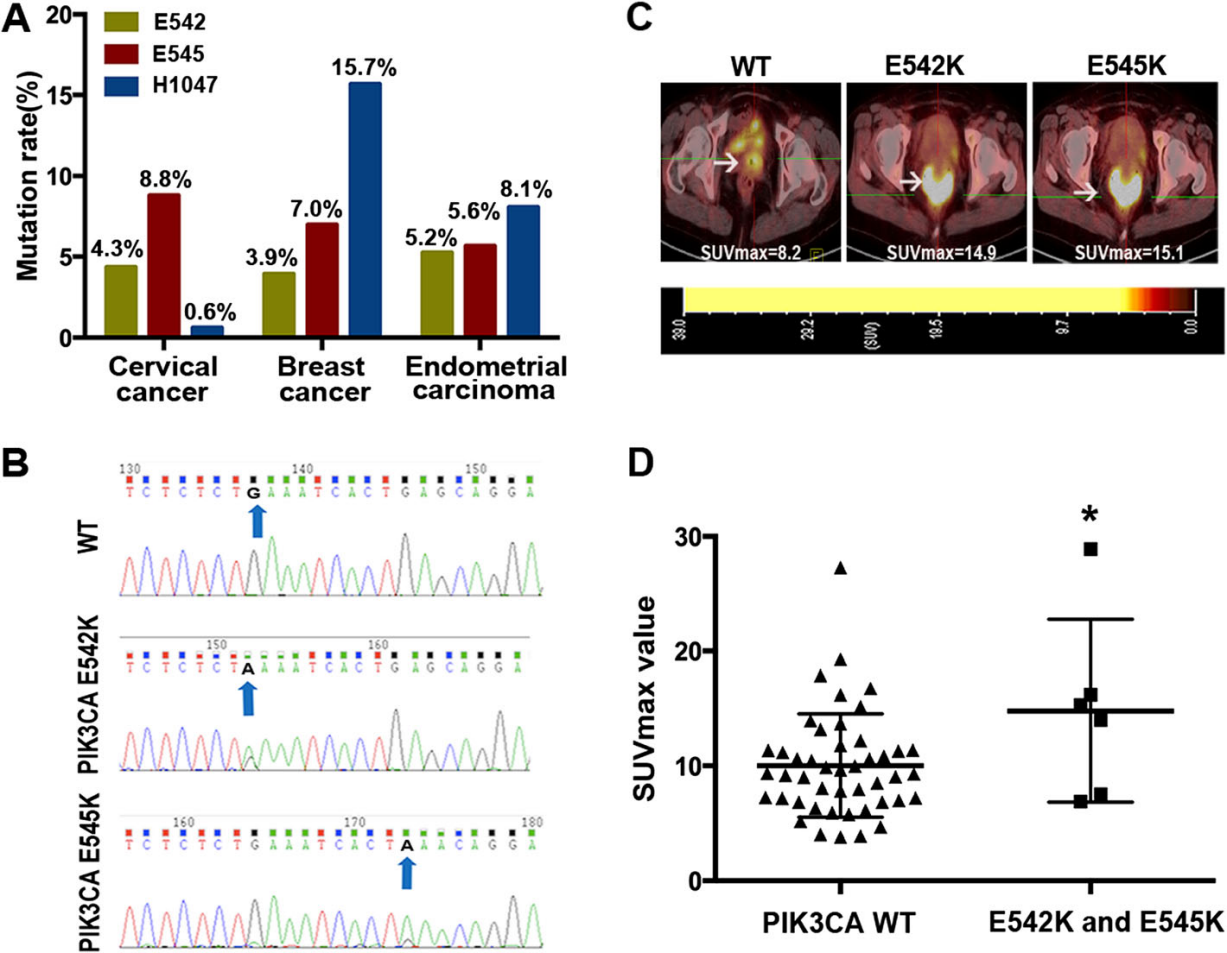
*PIK3CA* E542K, E545K mutation and its correlation with ^18^F-FDG PET/CT SUV_max_. **a** The mutation occurrence of *PIK3CA* E542, E545, and H1047 in cervical cancer, breast cancer, and endometrial carcinoma. **b** cDNA sequence of wild-type *PIK3CA* and mutated at E542K and E545K**. c** The representative ^18^F-FDG PET/CT imaging in patients with wild-type and mutant *PIK3CA*. **d** Statistical analysis of SUV_max_ in groups with wild-type and mutant *PIK3CA* (*n* = 52; *P* = 0.037)

*PIK3CA* mutations were detected in 146 of the total 990 patients with cervical cancer (146/990, 14.7%), including 132 mutations at exon 9 (132/990, 13.3%) and 14 mutations at exon 20 (20/990, 1.4%). The distribution of amino acid change is shown in Table 1. The frequency of E542 and E545 (exon 9, helical domain) mutation was significantly higher than that of H1047 (exon 20, kinase domain) (Fig. 1a, b). Different from cervical cancer, TCGA database indicated that the *PIK3CA* H1047 mutation was more common than that of E542 and E545 mutations in breast and endometrial carcinoma (Fig. 1a, b). According to Ciriello et al., among 817 invasive lobular breast cancer patients, 128 patients harbored *PIK3CA* mutations at H1047 (128/817, 15.7%), which was definitely predominant than that at E542 (32/817, 3.9%) and E545 (57/817, 7.0%) [22]. Similarly, the frequency of variants occurring at H1047 was higher than that at E542 and E545 in endometrial carcinoma (8.1% vs. 5.2% vs. 5.6%) [23]. The mutation distribution is presented in Table 1.

**Table 1:** *PIK3CA* mutation distribution in cervical cancer, breast cancer, and endometrial carcinoma

	Cervical cancer (*n* = 990)			Breast cancer (*n* = 817)			Endometrial carcinoma (*n* = 248)		
	Amino acid change	No. of cases	Frequency of variants (%)	Amino acid change	No. of cases	Frequency of variants (%)	Amino acid change	No. of cases	Frequency of variants (%)
Exon 9 E542	E542K	43	4.3	E542K	30	3.7	E542K	9	3.6
				E542G	2	0.2	E542A	2	0.8
							E542Q	1	0.4
							E542V	1	0.4
	Total	43	4.3	Total	32	3.9	Total	13	5.2
Exon 9 E545	E545K	84	8.5	E545K	54	6.6	E545K	10	4.0
	E545A	1	0.1	E542A	2	0.2	E545D	2	0.8
	E545D	1	0.1	E545R	1	0.1	E545A	1	0.4
	E545Q	1	0.1				E545G	1	0.4
	Total	87	8.8	Total	57	7.0	Total	14	5.6
Exon 20 H1047	H1047R	5	0.5	H1047R	116	14.2	H1047R	12	4.8
	H1047L	1	0.1	H1047L	11	1.3	H1047L	6	2.4
				H1047Y	1	0.1	H1047Y	1	0.4
							H1047Q	1	0.4
	Total	6	0.6	Total	128	15.7	Total	20	8.1

### Tumors with *PIK3CA* E542K and E545K mutations present higher SUVmax values in^18^F-FDG PET/CT scan

To evaluate the effect of *PIK3CA* mutation on glucose metabolism, we analyzed the SUV_max_ value in 52 patients, including 46 patients with wild-type *PIK3CA* and 6 patients with *PIK3CA* mutation at E542 and E545. The results showed that tumors with mutant *PIK3CA* exhibited higher SUV_max_ values (*P* = 0.037) (Fig. 1c, d).

### *PIK3CA* E542K and E545K mutations promote glucose metabolism and proliferation in cervical cancer cells

**Fig. 2:**
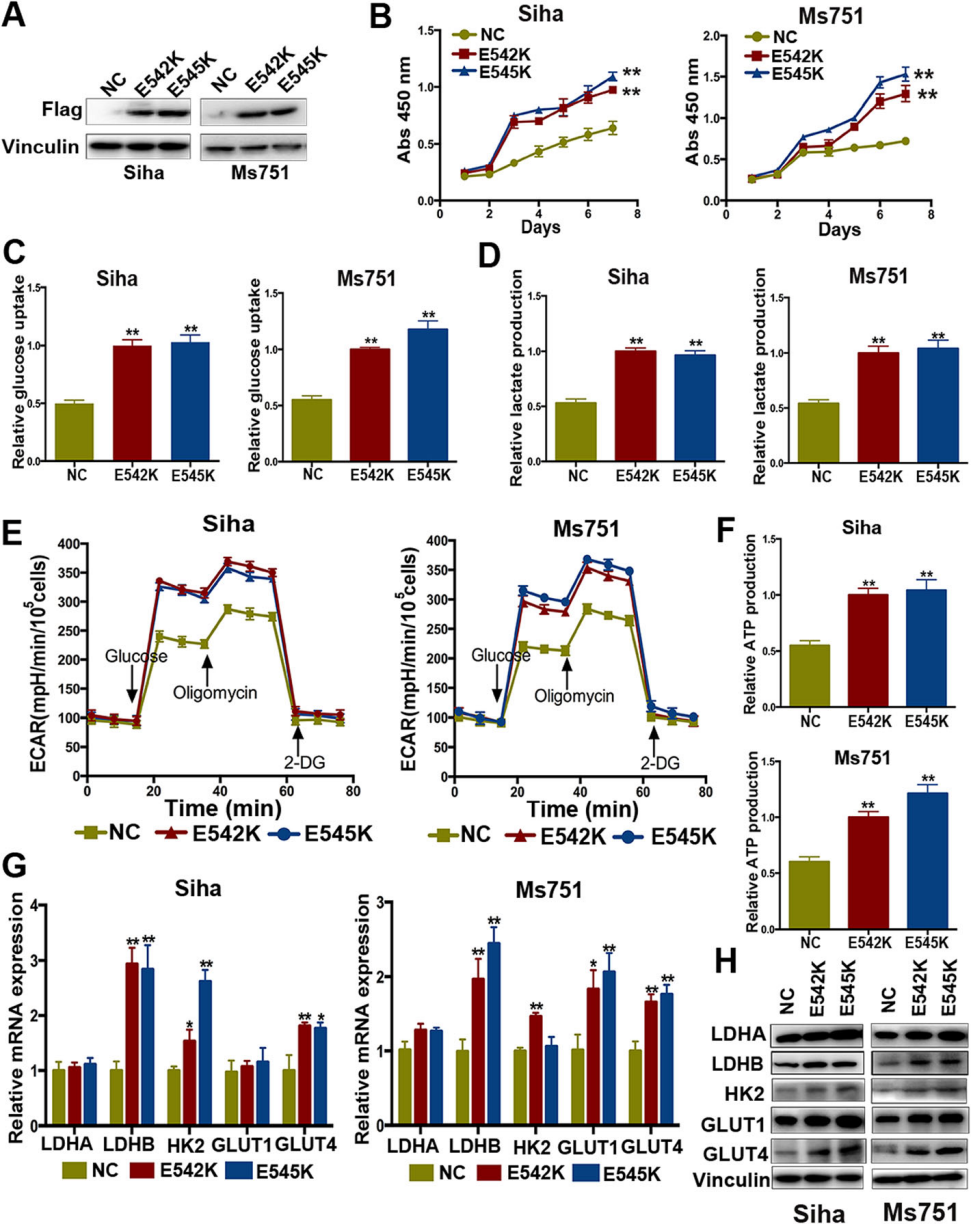
*PIK3CA* E542K and E545K mutations promote proliferation and glucose metabolism in cervical cancer cell. **a** Transfection of FLAG-tagged PI3K E542K and E545K in SiHa and MS751 cells. **b** Detection of proliferation by colony formation in SiHa and MS751 cells with wild-type and mutant *PIK3CA* (**P < 0.01). **c** The effect of *PIK3CA* E542K and E545K mutation on glucose uptake (**P < 0.01). **d** The effect of *PIK3CA* E542K and E545K mutation on lactate production (**P < 0.01). **e** ECAR analysis in SiHa and MS751 cells with wild-type and mutant *PIK3CA.* **f** *PIK3CA* E542K and E545K mutation affected ATP production (**P < 0.01). **g** Relative mRNA expression of key enzymes of glycolysis in SiHa and MS751 cells with wild-type and mutant *PIK3CA* (*P < 0.05; **P < 0.01). **h** The effect of *PIK3CA* E542K and E545K mutation on the expression level of key glycolytic enzyme

To further determine the function of PI3K E542K and E545K in cervical cancer, we introduced PI3K E542K and E545K cDNA into SiHa and MS751 cells and established SiHa/PI3K E542K, SiHa/PI3K E545K, MS751/PI3K E542K, and MS751/PI3K E545K cells stably expressing PI3K E542K and E545K cDNA (Fig. 2a). The results of the CCK8 assay indicated that *PIK3CA* E542K and E545K mutations significantly promoted cell proliferation in SiHa and MS751 cells (Fig. 2b). To evaluate the effect of *PIK3CA* E542K and E545K mutations on the alteration of glucose metabolism, we analyzed the level of glucose uptake and lactate production and found that cells with mutant *PIK3CA* had a higher turnover of glucose uptake and lactate production, indicating that *PIK3CA* E542K and E545K mutations enhanced the level of glycolysis in cervical cancer (Fig. 2c, d). ECAR (extracellular acidification rate) is another biomarker to assess glycolysis by measuring the level of lactate production. The results showed that *PIK3CA* E542K and E545K mutations increased ECAR in SiHa and MS751 cells (Fig. 2e). Furthermore, we examined the level of ATP production, a terminal indicator of glucose metabolism. As expected, ATP production was dramatically increased in SiHa and MS751 cells with mutant *PIK3CA* (Fig. 2f).

To explore the potential underlying mechanism of *PIK3CA* mutation-mediated glycolysis, key glycolytic enzymes were examined. Higher mRNA and protein expression of key enzymes associated with cell glycolysis (LDHA, LDHB, HK2, GLUT1, and GLUT4) was found in SiHa and MS751 cells harboring mutant *PIK3CA* (Fig. 2g, f), suggesting that *PIK3CA* E542K and E545K mutations promoted the glycolysis by the increased expression of key glycolytic enzymes. In addition, the expression levels of other glycolytic enzymes increased to varying degrees in SiHa and MS751 cells with *PIK3CA* E542K and E545K mutations (Additional file 2: Figure S1a and S1b). Taken together, *PIK3CA* E542K and E545K mutations enhance glucose metabolism and proliferation in cervical cancer cells.

### *PIK3CA* E542K and E545K mutations enhance proliferation and glucose metabolism by promoting the expression and nuclear accumulation of β-catenin in cervical cancer cells

**Fig. 3:**
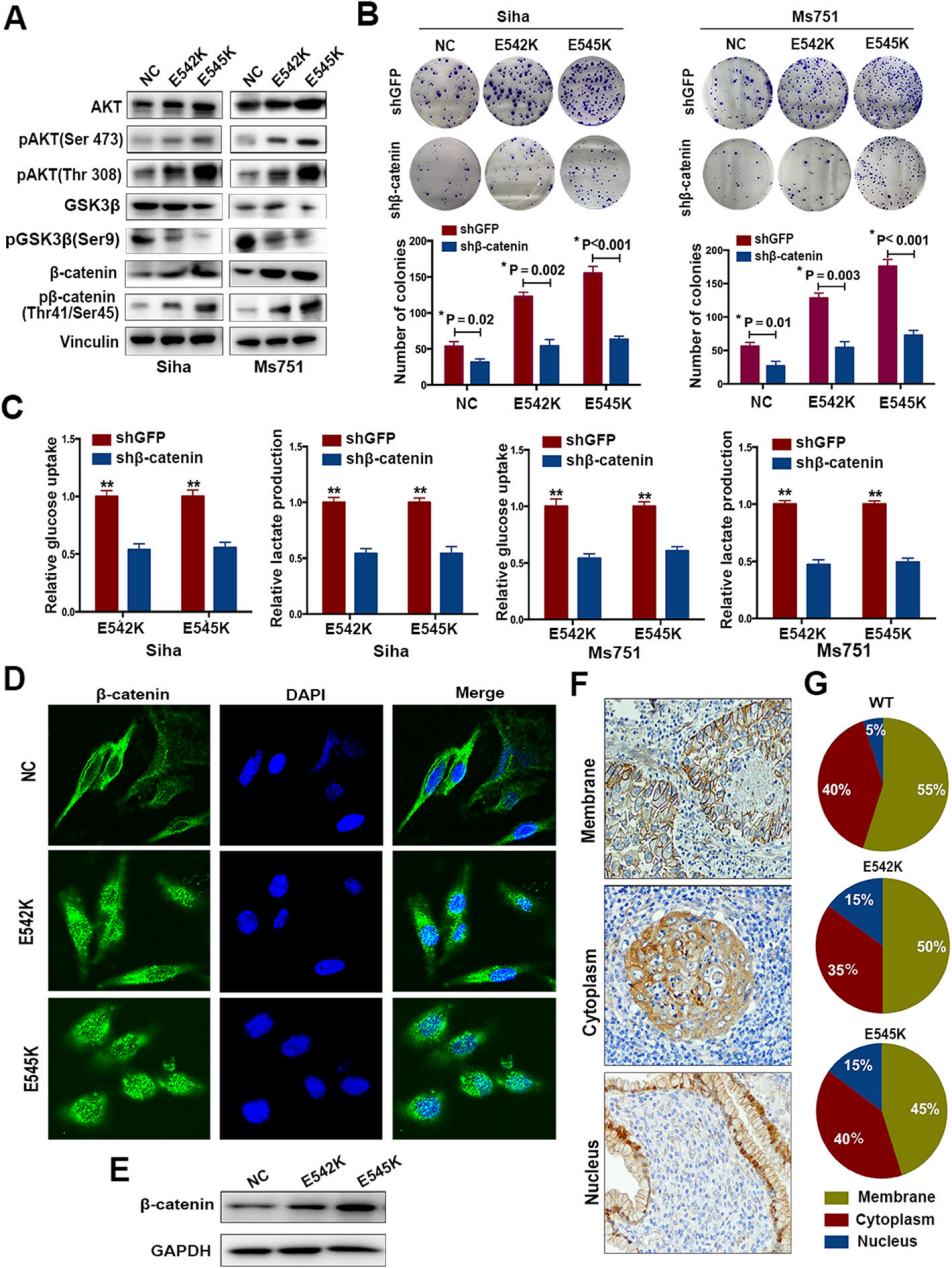
*PIK3CA* E542K and E545K mutations enhance the expression and nuclear accumulation of β-catenin. **a** The expression of AKT/GSK3 β/β-catenin in SiHa and MS751 cells with wild-type and mutant *PIK3CA*. **b** The effect of knocking down β-catenin on proliferation in SiHa and MS751 cells with wild-type and mutant *PIK3CA*. **c** The effect of knocking down β-catenin on glucose uptake and lactate production in SiHa and MS751 cells with wild-type and mutant *PIK3CA* (**P < 0.01). **d** The location of β-catenin was analyzed by immunofluorescence staining in SiHa cells. **e** The expression of β-catenin at nucleus in SiHa cells with wild-type and mutant *PIK3CA* by western blotting. **f** The representative imaging of β-catenin in membrane, cytoplasm, and nucleus by IHC in 60 patients with cervical cancer. **g** The corresponding proportion of β-catenin in membrane, cytoplasm, and nucleus in tissues of patients with wild-type and E542K, E545K mutant *PIK3CA*

**Fig. 4:**
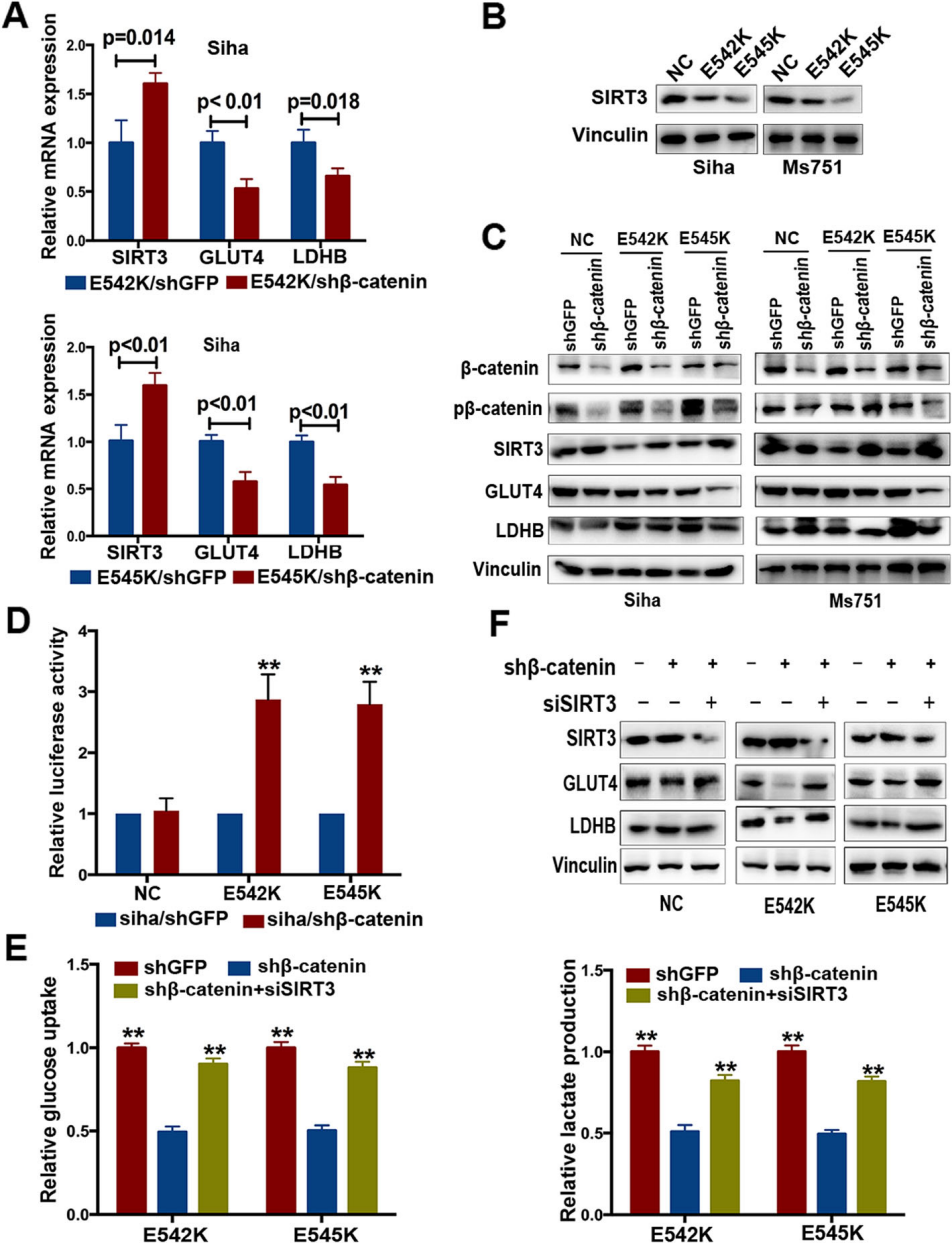
SIRT3 is a negative effector of β-catenin in regulating glucose metabolism. **a** Relative mRNA expression of SIRT3, GLUT4, and LDHB in SiHa cells with *PIK3CA* E542K and E545K mutations. **b** The expression of SIRT3 in SiHa and MS751 cells with wild-type and mutant *PIK3CA*. **c** Effect of knocking down β-catenin on the expression of SIRT3 and key glycolytic enzymes. **d** Relative SIRT3 promotor activity in SiHa cells with downregulation of β-catenin (**P < 0.01). **e** Knocking down SIRT3 rescued the effect of shβ-catenin on glucose uptake, lactate production (**P < 0.01). **f** Knocking down SIRT3 rescued the effect of shβ-catenin on key glycolytic enzymes

PI3K activates the AKT/GSK3β/β-catenin signaling pathway to regulate cell proliferation; therefore, we compared the expression level of AKT/GSK3β/β-catenin in SiHa and MS751 cells with wild-type and mutant *PIK3CA*. The increased activation of AKT/GSK3β/β-catenin was observed in SiHa and MS751 cells with *PIK3CA* E542K and E545K mutations compared to that in cells with wild-type *PIK3CA* (Fig. 3a). To further explore whether activated AKT/GSK3β/β-catenin was involved in regulating glucose metabolism and proliferation, lentiviruses carrying shRNA against β-catenin were transfected into SiHa and MS751 cells with wild-type and mutant *PIK3CA*. Colony formation analysis showed that the growth potential was suppressed in SiHa and MS751 cells with wild-type and mutant *PIK3CA*, but the degree of inhibition was more remarkable in cells with mutant *PIK3CA* (Fig. 3b). Similarly, the level of glucose uptake and lactate production significantly decreased following the downregulation of β-catenin in SiHa and MS751 cells harboring mutant *PIK3CA* (Fig. 3c). Moreover, the expression levels of GLUT4 and LDHB were markedly decreased in SiHa and MS751 cells with mutant *PIK3CA* following the knockdown of β-catenin expression (Fig. 4c), while LDHA, HK2, and GLUT1 did not show evident changes in expression (Additional file 3: Figure S2). Taken together, *PIK3CA* E542K and E545K mutations promote glucose metabolism and proliferation by inducing the AKT/GSK3β/β-catenin in cervical cancer cells.

The nuclear accumulation of β-catenin is involved in neoplastic transformation and tumor progression by regulating fat and glucose metabolism [24, 25]. The location of β-catenin is adversely regulated by GSK3β [26, 27]. Considering the high activation of AKT/GSK3β/β-catenin in cervical cancer cells with mutant *PIK3CA*, we inferred that *PIK3CA* E542K and E545K mutations might promote the nuclear accumulation of β-catenin in cervical cancer cells. First, we used immunofluorescence assays to compare the location of β-catenin in SiHa cells with wild-type and mutant *PIK3CA*. A significantly higher level of nuclear β-catenin expression was observed in SiHa cells with mutant *PIK3CA* (Fig. 3d). Second, the increased expression of β-catenin in the nucleus was confirmed in SiHa cells with mutant *PIK3CA* by western blot analysis (Fig. 3e). Furthermore, immunohistochemistry (IHC) was used to compare the location distribution of β-catenin in cervical cancer tissues from 40 patients with mutant *PIK3CA* and 20 patients with wild-type *PIK3CA*. Representative images of β-catenin in the membrane, cytoplasm, and nucleus are presented in Fig. 3f. Nuclear expression of β-catenin was found in 6 (15%) patients with mutant *PIK3CA*, which was more common than that in patients with wild-type *PIK3CA* (1/20), although this finding did not achieve statistical significance, likely due to the limited sample number (*P* = 0.247, chi-square test, Fig. 3g). Taken together, *PIK3CA* E542K and E545K mutations play a positive role in promoting the nuclear accumulation of β-catenin in cervical cancer cells.

### β-Catenin regulates glucose metabolism via the suppression of SIRT3 in cervical cancer cells with *PIK3CA* E542K and E545K mutations

To further explore the downstream molecular targets regulated by β-catenin, a series of genes involved in glucose metabolism were analyzed in SiHa cells with mutant *PIK3CA* after the downregulation of β-catenin. We found that the mRNA expression of SIRT3 was marked increased, while that of GLUT4 and LDHB significantly decreased with the downregulation of β-catenin, indicating that SIRT3, GLUT4, and LDHB might be regulated by β-catenin at the transcriptional level (Fig. 4a). Furthermore, we found that the protein expression level of SIRT3 in SiHa and MS751 cells with mutant *PIK3CA* was significantly lower than that in cells with wild-type *PIK3CA* (Fig. 4b), and the expression of SIRT3 increased following the knockdown of β-catenin expression (Fig. 4c). Further, correlation analysis indicated it was significantly negative associated with β-catenin in cells with SiHa E545K and MS751 E545K mutation (*P* < 0.05). Although it did not achieve statistical significance in cells with SiHa E542K and MS751 E542K mutation, it was still suggested that the expression of β-catenin was negatively correlated with that of SIRT3 (Additional file 4: Figure S3). SIRT3, a major mitochondrial deacetylase, mediates metabolic reprogramming and promotes the conversion to glycolysis [28]. Consequently, we inferred that SIRT3 might be involved in glucose metabolism in cervical cancer cells with mutant *PIK3CA*, which was adversely regulated by β-catenin. A luciferase assay was used to determine whether SIRT3 promotor was regulated by β-catenin in cervical cancer cells with *PIK3CA* E542K and E545K mutations. The activity of the SIRT3 promotor markedly increased in SiHa cells with *PIK3CA* E542K and E545K mutations following the downregulation of β-catenin, whereas this increase was not evident in SiHa cells with wild-type *PIK3CA*, suggesting that β-catenin had a negative impact on the promoter activity of SIRT3 in cervical cancer cells with mutant *PIK3CA* (Fig. 4d). Moreover, the decreased glucose uptake and lactate production due to the downregulation of β-catenin was reversed by transfection with SIRT3 siRNA in SiHa cells with mutant *PIK3CA* (Fig. 4e). Similarly, the decreased expression of GLUT4 and LDHB was also reversed (Fig. 4f). Taken together, these results demonstrate that SIRT3 functions as a downstream effector of β-catenin in regulating glucose metabolism and glycolysis activity.

### *PIK3CA* E545K mutation enhances glucose metabolism and proliferation in cervical cancer xenografts

**Fig. 5:**
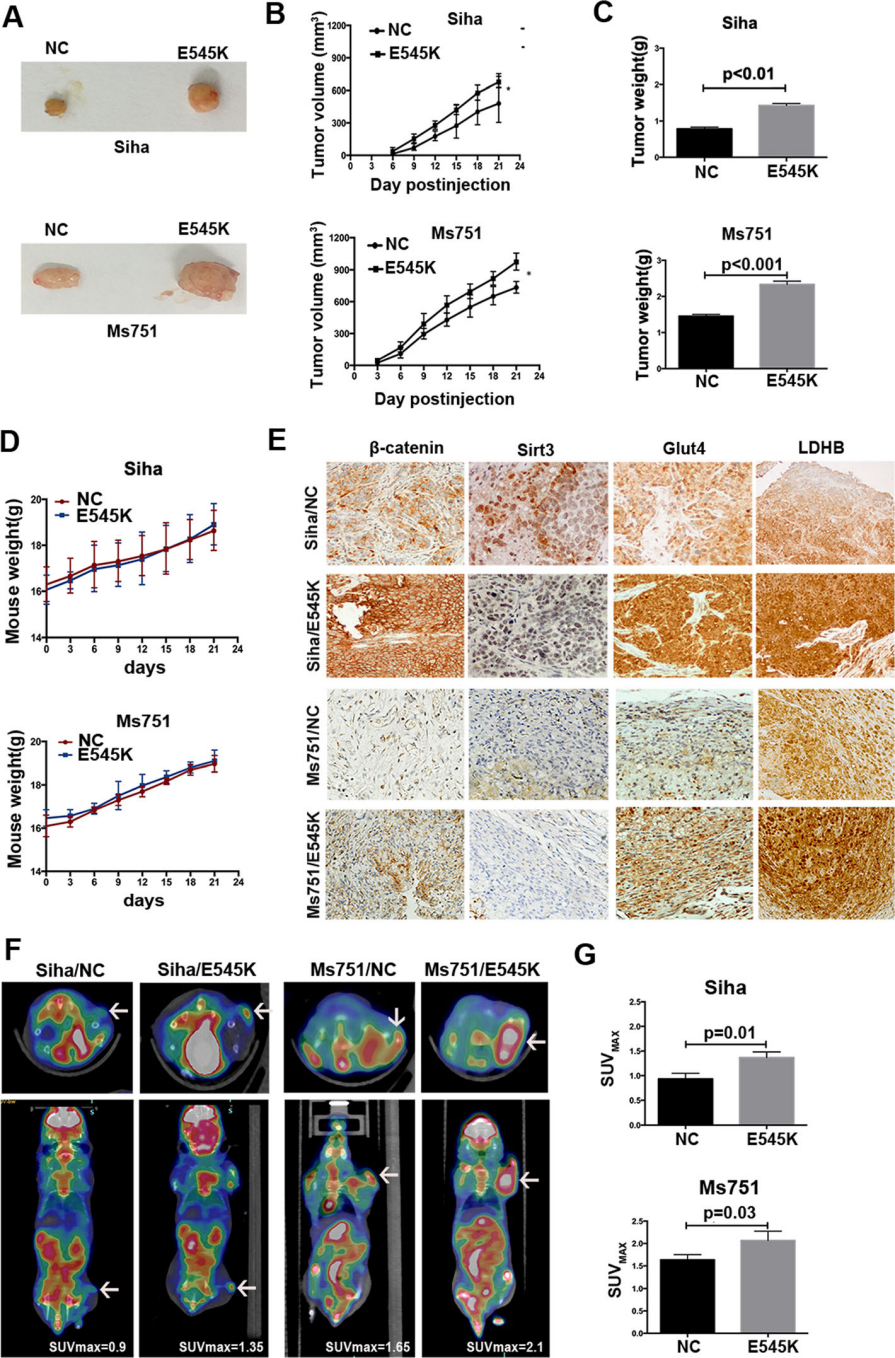
*PIK3CA* E542K and E545K mutations positively regulate proliferation and glucose metabolism in xenograft mice. **a** The tumors were separated from the xenograft mice with SiHa and MS751 cells harbored wild type and E545K mutant *PIK3CA*. **b** The growth curve of xenograft tumors from SiHa and MS751 cells with wild type and E545K mutant *PIK3CA*. **c** Tumor weight of xenograft mice with SiHa and MS751 cells harbored wild type and E545K mutant *PIK3CA*. **d** The weight of xenograft mice with SiHa and MS751 cells harbored wild type and E545K mutant *PIK3CA*. **e** Immunohistochemical staining of xenograft tumor tissues. **f** Representative ^18^F-FDG microPET/CT imaging of tumor-bearing mice. The tumors are indicated with arrows. **g** The tumor SUV_max_ in xenograft mice with wild type and mutant *PIK3CA*

Considering the similar effect of *PIK3CA* E542K and E545K mutations on glucose metabolism and proliferation, we confirmed the effects of *PIK3CA* E545K mutation in cervical cancer xenografts. SiHa and MS751 cells with wild-type and mutant E545K *PIK3CA* were subcutaneously injected into nude mice. Consistently, the growth of tumors with cells harboring mutant *PIK3CA* was significantly faster than that of tumors with wild-type *PIK3CA* (Fig. 5a, b). Additionally, the tumors of cells with mutant *PIK3CA* outweighed those of cells with wild-type *PIK3CA* (Fig. 5c), and injection had no effect on the weight of nude mice (Fig. 5d). The above results indicate that *PIK3CA* E545K mutation promotes proliferation in xenograft models.

We then evaluated the role of *PIK3CA* E545K mutation in glucose metabolism. Small animal imaging was performed to determine the uptake of ^18^F-FDG in a xenograft mouse model. The results showed that tumors with mutant *PIK3CA* exhibited higher ^18^F-FDG intake, indicating a higher level of glucose uptake in tumors with *PIK3CA* E545K mutation (Fig. 5f, g). Furthermore, we analyzed the expression of β-catenin, SIRT3, GLUT4, and LDHB by immunohistochemistry in xenografts with wild-type and mutant *PIK3CA*. The expression levels of β-catenin, GLUT4, and LDHB in xenograft models with *PIK3CA* E545K were enhanced, while that of SIRT3 was decreased compared with those in the corresponding controls, which was consistent with that in cervical cancer cells in vitro (Fig. 5e). Thus, *PIK3CA* E545K mutation has a positive impact on glucose metabolism in cervical cancer xenografts.

### The expression analysis of β-catenin, SIRT3, GLUT4, and LDHB in tissues of patients with cervical cancer

**Fig. 6:**
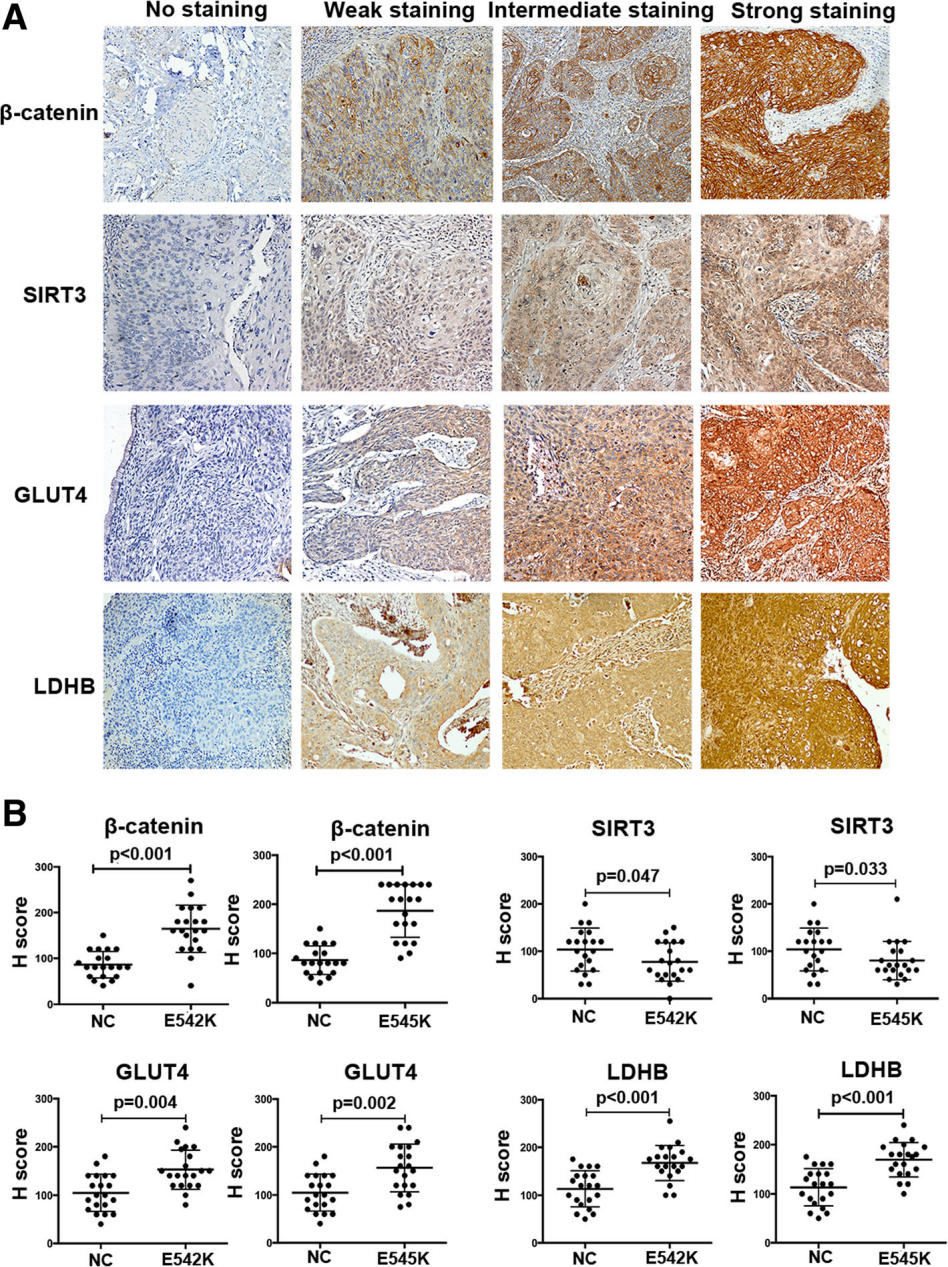
Immunohistochemical analyses of β-catenin, SIRT3, GLUT4, and LDHB expression in tissues of patients with cervical cancer. **a** Representative pictures of β-catenin/SIRT3 and GLUT4, LDHB in negative, weak staining, intermediate staining, and strong staining. **b** The statistical analysis of *H* score of β-catenin/SIRT3 and GLUT4, LDHB in cervical cancer tissues

To further verify the association between β-catenin and SIRT3 in glucose metabolism in vivo, we compared the expression of β-catenin, SIRT3, GLUT4, and LDHB in patients with cervical cancers harboring wild-type *PIK3CA* (*n* = 20), *PIK3CA* E542K mutation (*n* = 20), and *PIK3CA* E545K mutation (*n* = 20) by IHC. The expression of these proteins was significantly different between tumors harboring wild-type and mutant *PIK3CA* (*P* < 0.05). Correlation analysis showed that the expression of SIRT3 was negatively associated with β-catenin in tumors with *PIK3CA* E542K and E545K mutation, although the curve did not show a good fit (Additional file 5: Figure S4). In addition, the expression of key glycolytic enzymes GLUT4 and LDHB was remarkably higher in tissues with mutant *PIK3CA*, suggesting that glycolytic metabolism was enhanced in tumors with mutant *PIK3CA* (Fig. 6).

## Discussion

Metabolic reprogramming has been recognized as a hallmark of cancer in recent centuries [29]. To adapt well to nutrient-deficient circumstances, cancer cells activate some metabolic pathways, such as NADPH and ATP, to enhance the production of energy for cell growth and proliferation. However, some oncogenic molecules will be activated and exert positive effects on proliferation and survival [30–32]. The aberrant activation of the PI3K/AKT pathway promotes glucose uptake and glycolysis via the regulation of effectors, such as GLUT1 and GLUT4 [18, 33, 34]. A previous study demonstrated that *PIK3CA* was the most common mutated gene in cervical cancer. Thus, it was necessary to explore the effect of *PIK3CA* mutation on proliferation and metabolism alterations in cervical cancer.

^18^F-FDG PET/CT, an imaging examination based on tumor metabolic activity, has been widely used in the clinic for cancer diagnosis, disease monitoring, and evaluation of treatment response [35–37]. Generally, the elevated SUV_max_ value indicates enhanced glucose uptake and glycolysis activity. In the present study, we found that the level of glucose metabolism in cervical cancer patients with mutant *PIK3CA* was dramatically higher than that of patients with wild-type *PIK3CA* based on the increased SUV_max_ value detected by ^18^F-FDG PET/CT. Similar results were obtained in xenograft models. Enhanced proliferation and glucose metabolism was further confirmed in cervical cancer cells and xenograft models with mutant *PIK3CA*. These results indicate that *PIK3CA* E542K and E545K mutations play a vital role in promoting glycolysis and proliferation in vitro and in vivo.

In terms of mechanism, the AKT/GSK3β/β-catenin signaling pathway is involved in regulating glucose metabolism. AKT, a well-studied effector of PI3K, is an important driver of tumor glycolytic activity, which enables rapid ATP generation to maintain energetic metabolism. In response to insulin stimulation, the rapid activation of AKT2 increases the expression and translocation of GLUT4, which is essential for insulin-stimulated glucose uptake [18, 34, 38, 39]. AKT signaling also induces the activation of FOXO transcription factors, resulting in a series of downstream transcriptional changes to increase glycolytic capacity [40]. Moreover, the PI3K/mTOR signaling pathway induces the high expression of metabolic gene regulatory networks [41]. Furthermore, AKT negatively regulates GSK3, a type of glycogen synthase, which is responsible for the glycogen synthesis. The inhibition of GSK3 leads to alterations of glucose metabolism via the upregulation of HK2 [42]. Mounting evidence suggested that β-catenin, as an effector of AKT/GSK3, had a close correlation with glucose metabolism. In colorectal cancer cell, β-catenin is translocated to the nucleus and interacts with HIF1α instead of TCF to adjust metabolic patterns during periods of hypoxia [43]. Consequently, we proposed that GSK3β/β-catenin is likely involved in regulating glucose metabolism and proliferation in cervical cancer with mutant *PIK3CA*. In the present study, the high expression and nuclear translocation of β-catenin was observed in cervical cancer cells and tumor tissues with mutant *PIK3CA*. The downregulation of β-catenin rapidly decreased the proliferation, glucose uptake, and lactate production of tumors with mutant *PIK3CA*. The expression of GLUT4 and LDHB were abated following the reduction of β-catenin. Altogether, these results suggest that β-catenin is a vital molecule involved in regulating proliferation and glucose metabolism in cervical cancer cells with mutant *PIK3CA*.

The sirtuin family has been associated with metabolic regulation in recent years. SIRT3, a member of the sirtuin family, is a type of NAD^+^-dependent mitochondrial deacetylase, which is involved in negatively regulating glucose metabolism, superoxide levels, and total cellular ATP levels [44]. SIRT3 promotes cellular metabolism and growth by destabilizing HIF1α, a well-known transcription factor regulating glycolysis-related gene expression [45]. The results of the present study suggested that SIRT3 is a direct downstream effector of β-catenin in regulating glucose metabolism. Mechanistically, β-catenin inhibited the activity of the SIRT3 promotor and decreased the expression of SIRT3 at the transcriptional level. Furthermore, the expression of SIRT3 was adversely related to β-catenin in cervical cancer tissues and xenograft models by IHC.

**Fig. 7:**
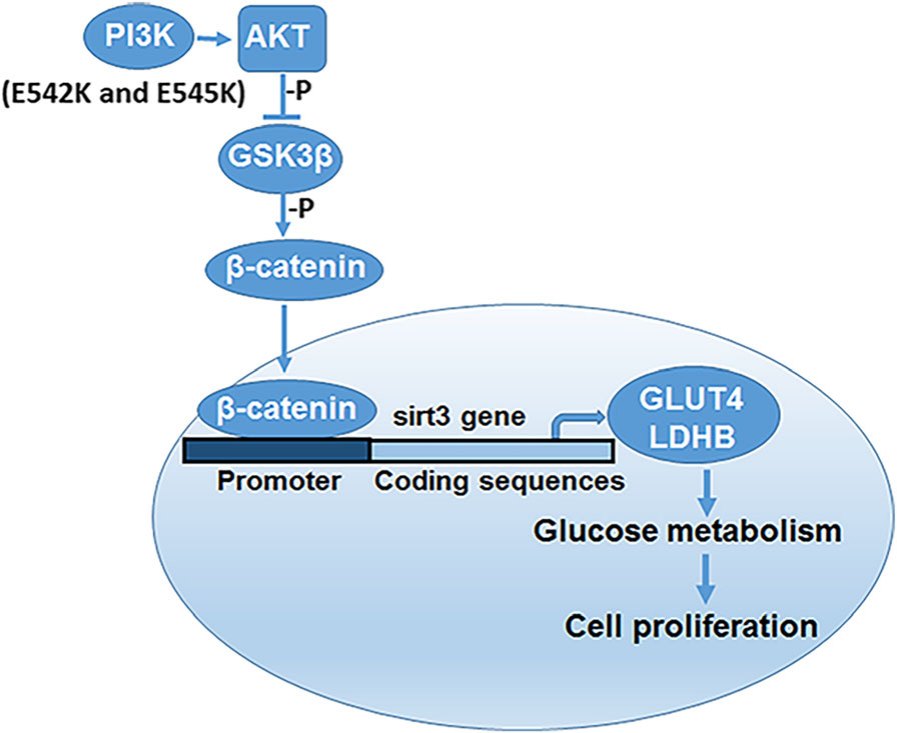
Mechanism model of *PIK3CA* E542K and E545K mutation-mediate regulation of proliferation and glucose metabolism via the β-catenin/SIRT3 in cervical cancer

In conclusion, we demonstrate that *PIK3CA* E542K and E545K mutations induce glycolysis in cervical cancer cells through the induction of the β-catenin/SIRT3 signaling pathway (Fig. 7), which offers important implications for the underlying mechanism of *PIK3CA* E542K and E545K mutation-mediated glycolysis and proliferation, but also provide the new insights into the development of therapeutic approaches using *PIK3CA* E542K and E545K mutations as a target to prevent metastasis in various cancers, including cervical cancer.

## Conclusions

The proliferative and glycolytic potential was enhanced in cervical cancer with *PIK3CA* E542K and E545K mutations in vivo and in vitro. Mechanistically, *PIK3CA* E542K and E545K mutations promote the expression and nuclear accumulation of β-catenin, which negatively regulates the expression of SIRT3. These findings provide evidence that the *PIK3CA* E542K and E545K/β-catenin/SIRT3 signaling axis regulates glucose metabolism and proliferation and supply new evidence for the development of therapeutic targets to prevent tumor growth and recurrence.

## Additional files

Additional file 1: **Table S1.** The primer sequences are used for qRT-PCR detection. (DOCX 15 kb)

Additional file 2: **Figure S1.** Relative mRNA expression of other glycolytic enzymes in SiHa and MS751 cells with wild-type and mutant *PIK3CA*. A: Relative mRNA expression of other key glycolytic enzymes in SiHa cells with wild-type and mutant *PIK3CA*. B: Relative mRNA expression of other key glycolytic enzymes in MS751 cells with wild-type and mutant *PIK3CA*. (TIF 1495 kb)

Additional file 3: **Figure S2.** The expression of other key glycolytic enzymes after knocking down the expression of β-catenin. (TIF 1169 kb)

Additional file 4: **Figure S3.** The correlation analysis between β-catenin and SIRT3 by western blotting in SiHa and MS751 cells with wild-type and mutant *PIK3CA*. (TIF 682 kb)

Additional file 5: **Figure S4.** The correlation analysis between β-catenin and SIRT3 in cervical cancer tissues with wild-type and mutant *PIK3CA*. (TIF 457 kb)

## Abbreviations

ATCC: American Type Culture Collection
ATP: Adenosine triphosphate
ECAR: Extracellular acidification rate
GLUT1: Glucose transporter 1
GLUT4: Glucose transporter 4
HK2: Hexokinase 2
IHC: Immunohistochemistry
LDHA: Lactate dehydrogenase A
LDHB: Lactate dehydrogenase B
PI3K: Phosphatidylinositol 3-kinase
PVDF: Polyvinylidene fluoride
