# Supplementary material for: The PIK3CA E542K and E545K mutations promote glycolysis and proliferation via induction of the β-catenin/SIRT3 signaling pathway in cervical cancer
Source: J Hematol Oncol. 2018 Dec 14;11:139. doi: 10.1186/s13045-018-0674-5 (PMC6293652; doi:10.1186/s13045-018-0674-5)
Supplement: Supplementary file 1 — Table S1. The primer sequences are used for qRT-PCR detection. (DOCX 15 kb) [file 13045_2018_674_MOESM1_ESM.docx]

**Table S1.** The primer sequences are used for qRT-PCR detection.

| **Gene name** | **Primer sequnces** |
| --- | --- |
| **GLUT1-F** | **5-CTTTGTGGCCTTCTTTGAAGT-3** |
| **GLUT1-R** | **5-CCACACAGTTGCTCCACAT-3** |
| **HK2-F** | **5-GATTGTCCGTAACATTCTCATCGA-3** |
| **HK2-R** | **5-TGTCTTGAGCCGCTCTGAGAT-3** |
| **G6P-F** | **5-GGTACACAGGCAAGACCATC-3** |
| **G6P-R** | **5-GTTTTGGCAATGTGAGTTCC-3** |
| **PFKL-F** | **5-CACAGGTGCCAACATCTTCCGCA-3** |
| **PFKL-R** | **5-TCATGTCGGTGCCGCAGAAGTCG-3** |
| **ALDOA-F** | **5-GTTATCAAATCCAAGGGCGGTGTT-3** |
| **ALDOA-R** | **5-AGTCAGCTCCGTCCTTCTTGTAC-3** |
| **ALDOB-F** | **5-CACCATTCAAGGGCTTGATGGCCT-3** |
| **ALDOB-R** | **5-TTCCTGGATAGCGAGGCTGGAT-3** |
| **PGK1-F** | **5-CAAGGTTAAAGCCGAGCCAGCCAA-3** |
| **PGK1-R** | **5-GCCTTCTGTGGCAGATTGACTCC-3** |
| **PGAM1-F** | **5-ATGATGTCCCACCACCTCCGAT-3** |
| **PGAM1-R** | **5-ATCCTTCAGACTCTCACAGGAG-3** |
| **ENO1-F** | **5-GCTCCGGGACAATGATAAGACTCG-3** |
| **ENO1-R** | **5-CTGTTCCATCCATCTCGATCATC-3** |
| **ENO2-F** | **5-TGAAGGCAGTGGACCACATCAACT-3** |
| **ENO2-R** | **5-AGAGACACACCCAGGATGGCATT-3** |
| **PKM2-F** | **5-CAAAGGACCTCAGCAGCCATGTC-3** |
| **PKM2-R** | **5-GGGAAGCTGGGCCAATGGTACAGA-3** |
| **LDHA-F** | **5-TGGAGATTCCAGTGTGCCTGTATGG-3** |
| **LDHA-R** | **5-CACCTCATAAGCACTCTCAACCACC-3** |
| **LDHB-F** | **5-GGAAGGAAGTGCATAAGATGGTGG-3** |
| **LDHB-R** | **5-CCCCTTTACCATTGTTGACACG-3** |
| **PKM1-F** | **5-CTATCCTCTGGAGGCTGTGC-3** |
| **PKM1-R** | **5-CCATGAGGTCTGTGGAGTGA-3** |
| **SIRT3-F** | **5-AGCCCTCTTCATGTTCCGAAGTGT-3** |
| **SIRT3-R** | **5-TCATGTCAACACCTGCAGTCCCTT-3** |
| **Actin-F** | **5-CTACGTCGCCCTGGACTTCGAGC-3** |
| **Actin-R** | **5-GATGGAGCCGCCGATCCACACGG-3** |
