# Supplementary figures and images for: The PIK3CA E542K and E545K mutations promote glycolysis and proliferation via induction of the β-catenin/SIRT3 signaling pathway in cervical cancer
Source: J Hematol Oncol. 2018 Dec 14;11:139. doi: 10.1186/s13045-018-0674-5 (PMC6293652; doi:10.1186/s13045-018-0674-5)

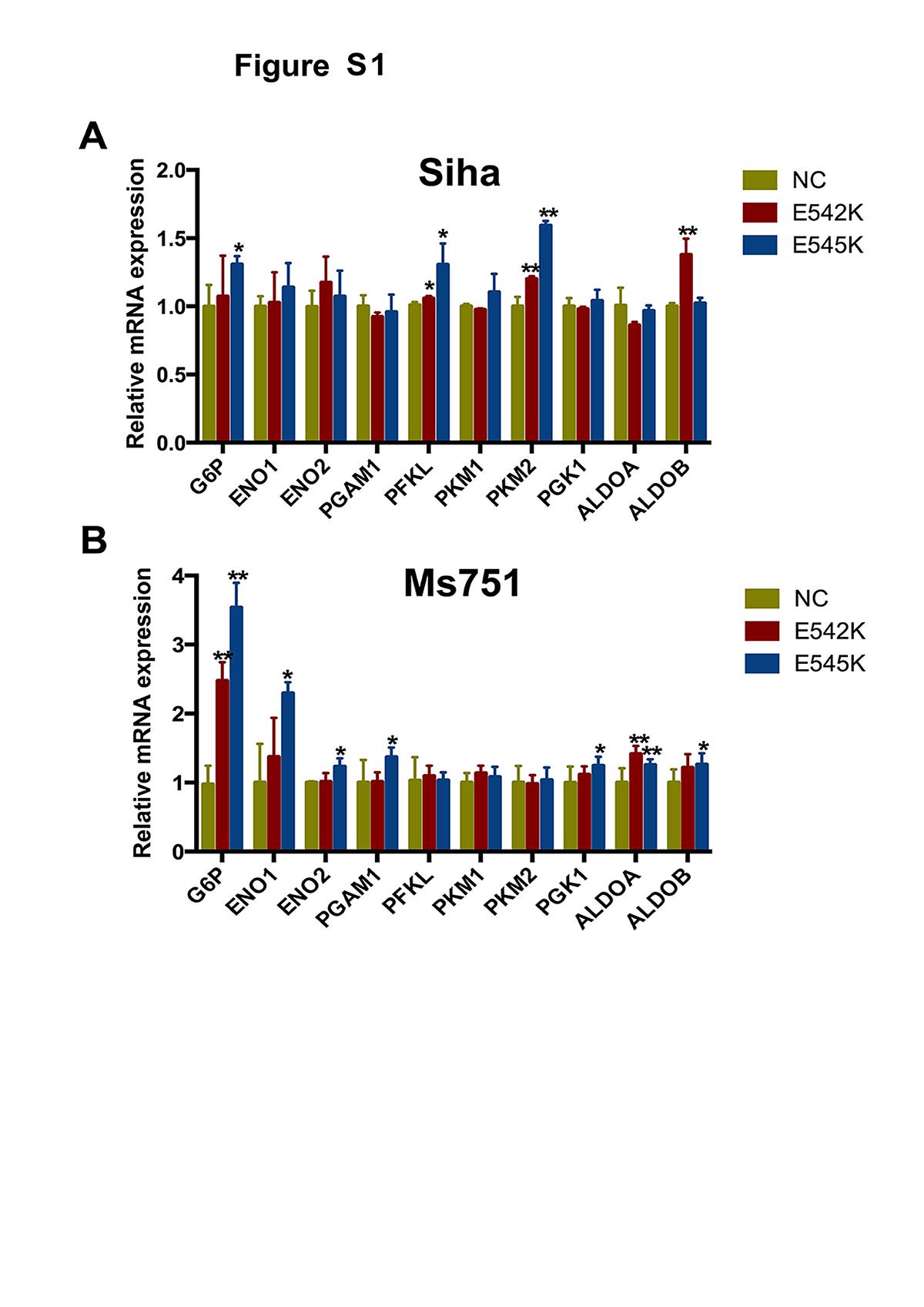

Supplement: Supplementary file 2 — Figure S1. Relative mRNA expression of other glycolytic enzymes in SiHa and MS751 cells with wild-type and mutant PIK3CA. A: Relative mRNA expression of other key glycolytic enzymes in SiHa cells with wild-type and mutant PIK3CA. B: Relative mRNA expression of other key glycolytic enzymes in MS751 cells with wild-type and mutant PIK3CA. (TIF 1495 kb) [file 13045_2018_674_MOESM2_ESM.tif]

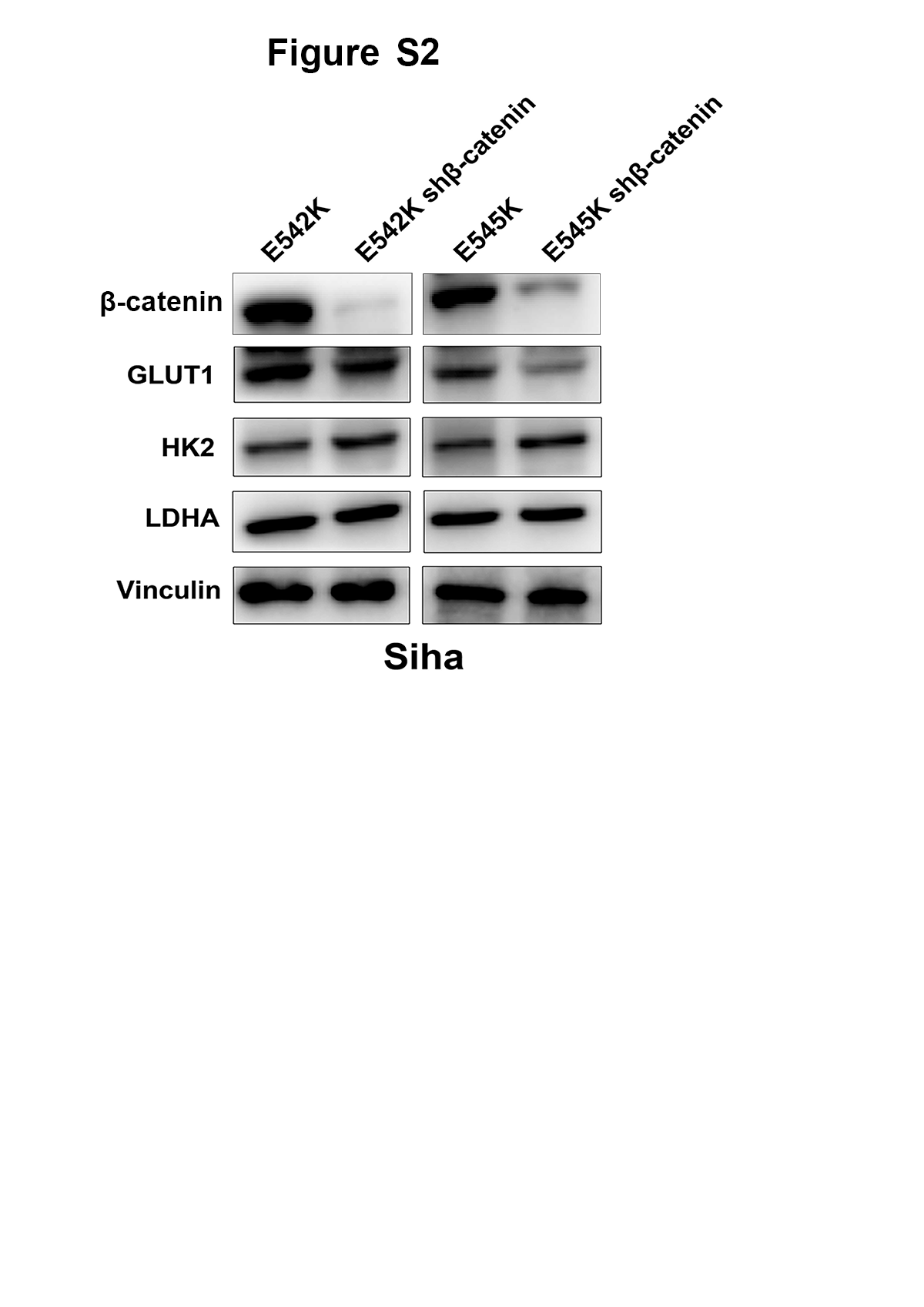

Supplement: Supplementary file 3 — Figure S2. The expression of other key glycolytic enzymes after knocking down the expression of β-catenin. (TIF 1169 kb) [file 13045_2018_674_MOESM3_ESM.tif]

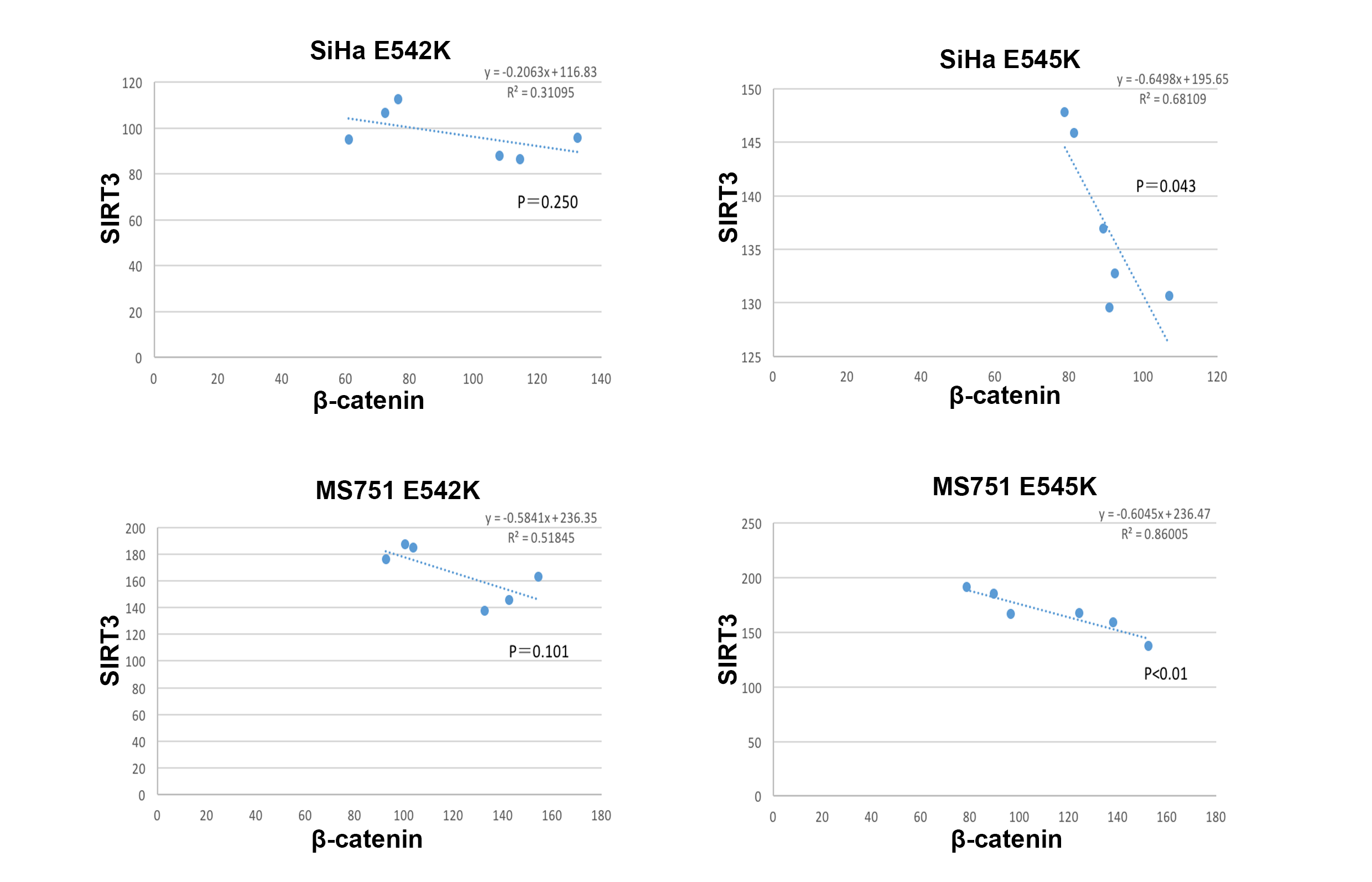

Supplement: Supplementary file 4 — Figure S3. The correlation analysis between β-catenin and SIRT3 by western blotting in SiHa and MS751 cells with wild-type and mutant PIK3CA. (TIF 682 kb) [file 13045_2018_674_MOESM4_ESM.tif]

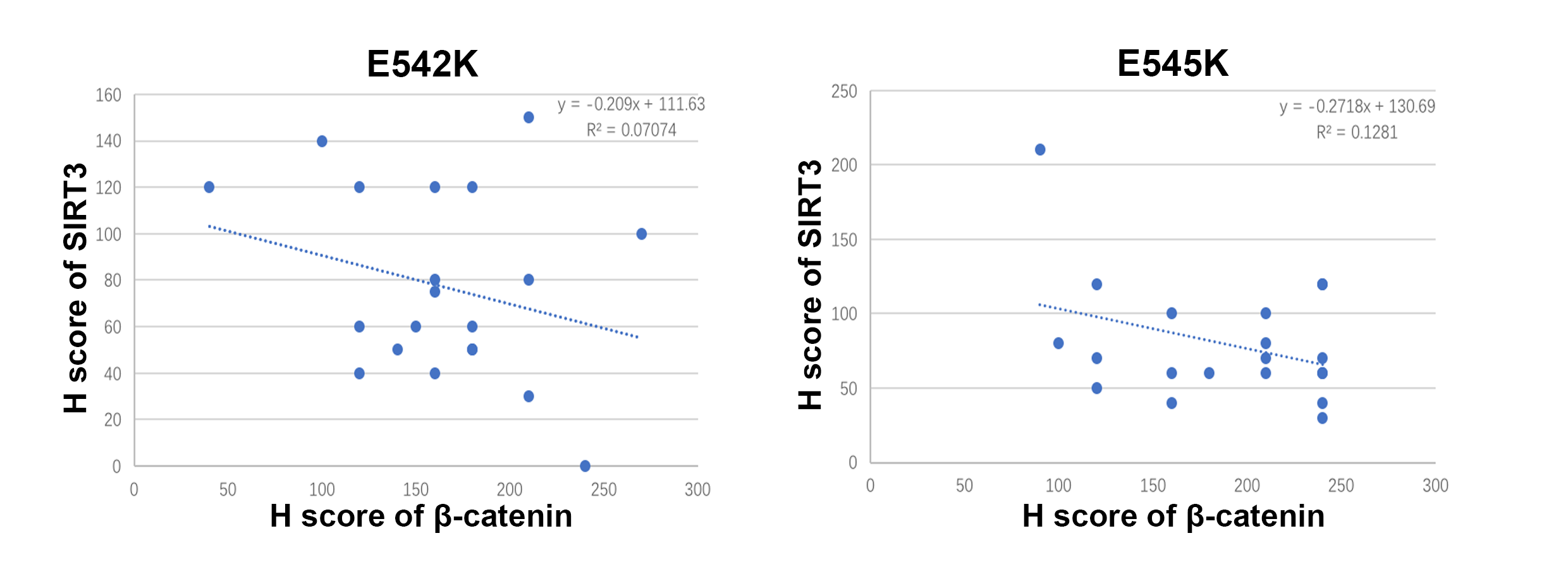

Supplement: Supplementary file 5 — Figure S4. The correlation analysis between β-catenin and SIRT3 in cervical cancer tissues with wild-type and mutant PIK3CA. (TIF 457 kb) [file 13045_2018_674_MOESM5_ESM.tif]
